# Hope for “Continued Vitality”: Qualitative Study of Adults With Traumatic Brain Injury and Low Mood on Their Rehabilitation

**DOI:** 10.3389/fresc.2022.848575

**Published:** 2022-02-22

**Authors:** Adora Chui, Katie N. Dainty, Bonnie Kirsh, Deirdre R. Dawson, Heather Colquhoun

**Affiliations:** ^1^Rehabilitation Sciences Institute, University of Toronto, Toronto, ON, Canada; ^2^Rotman Research Institute, Toronto, ON, Canada; ^3^Institute of Health Policy Management and Evaluation, University of Toronto, Toronto, ON, Canada; ^4^North York General Hospital, Toronto, ON, Canada; ^5^Department of Occupational Science and Occupational Therapy, University of Toronto, Toronto, ON, Canada

**Keywords:** person-centered care, qualitative study, traumatic brain injury, depression, rehabilitation

## Abstract

**Objective:**

Depression is highly comorbid with traumatic brain injury (TBI) with often complex and interacting symptomology that contributes to the experience of disability. Comorbid depression results in poorer TBI rehabilitation and downstream participation outcomes yet perspectives of this group regarding person-centered care is unknown.

**Purpose:**

This study aimed to explicate the perspectives of persons with TBI and depression on their values, preferences, and desired outcomes for optimal rehabilitation.

**Methods:**

A qualitative descriptive approach was taken. Thirteen adults [mean age: 40.5 (standard deviation 9.8)] diagnosed with TBI and with self-reported low mood were recruited through convenience sampling. Participants were predominantly female (*n* = 12) with concussion/mild TBI and at least 6 months post-injury. One-on-one, semi-structured interviews were conducted by phone with Canadian participants (March-May 2020). Interviews were transcribed; data were analyzed thematically by two researchers and the thematic map refined by the research team.

**Results:**

Three themes were identified on values, preferences, and desired outcomes in person-centered care. Participants valued “validation” from healthcare providers and the health system to feel seen and believed about their conditions and concerns. They preferred for healthcare providers to “share the burden of managing care” through improved interactions and better access to concussion care. Participants expressed that “meaningful outcomes” were to be symptom free, to resume valued life activities, and to be able to adapt/be resilient. The latter indicated hope for “continued vitality” for life participation despite past and ongoing challenges.

**Conclusions:**

Many adults with TBI and self-identified low mood expressed rehabilitation experiences that were invalidating. Their identified values, preferences, and desired outcomes provide directions for better person-centered care by healthcare providers and health systems to support participation.

## Introduction

Depression is a comorbidity which is highly prevalent in the traumatic brain injury (TBI) population (1), yet the rehabilitation experiences and participation outcomes of individuals with TBI and depression have not been a focus of research to date. Representation from this group is lacking in TBI clinical practice guidelines (2) which are evidence-based tools intended to improve rehabilitation and patient outcomes (3, 4), but which poorly incorporate patient values and preferences within treatment recommendations (5–8). Guidelines rarely address comorbid conditions (5, 9–11) and are predominantly disease-centered instead of person-centered (5, 12, 13) despite knowledge that person-centered care improves treatment outcomes, medication adherence, patient satisfaction, and quality of care (14, 15).

For individuals with TBI and depression, only a minority receive appropriate mood intervention (16) as comorbidities like psychiatric diagnoses often preclude access to existing services (17). There are compelling reasons to treat depression during TBI rehabilitation: having depressive symptoms likely results in slower recovery (18), and comorbid mental health is associated with lower quality of care (19) and increased cost and complexity (20). Providing person-centered care to individuals with TBI and depression—care that is “closely congruent with and responsive to patients' wants, needs, and preferences” (21)—may mitigate some of the shortcomings faced by underrepresented and/or comorbid groups (22, 23). However, for adults with TBI and depression, their values, preferences, and desired outcomes for rehabilitation, including participation, are unknown. Healthcare providers (HCPs) should ideally be giving person-centered care, “ensuring that patient values guide all clinical decisions” (24). Further, “a “good” outcome must be defined in terms that are meaningful and valuable to the individual patient” (25) and be broader than merely clinical/medical endpoints (26).

We focused on patient perspectives because patients have distinct values and preferences regarding their interactions with HCPs (27), and because their rehabilitation experiences are influenced by HCP attitudes and by the quality of their interactions (28, 29). Findings from this study will inform the provision of person-centered rehabilitation to adults with TBI and depression, so to bolster their participation in personally meaningful activities and life situations. “Person” in person-centered care indicates an ethical orientation toward individuals as human beings and social creatures (30, 31) with preferences beyond the clinical realm (26) and specific healthcare context (32). However, we also use the term “patient” when referring to a healthcare setting to distinguish the views of these individuals from HCPs—the latter holds the balance of power when care is not person-centered (33); participation as the goal of rehabilitation requires understanding patient perspectives. Therefore, the purpose of this study is to explicate the perspectives of persons living with TBI and depression on values, preferences, and desired outcomes related to their rehabilitation.

## Methods

We used a qualitative descriptive approach which is appropriate to understand little-known phenomena and lived experiences from the stance of the participants (34). From the philosophical position of naturalistic inquiry, qualitative description allows participants to describe the meaning of the phenomenon—here, the care of adults with TBI and depression—after which the analytic work produces a literal understanding and then an interpretation of those realities (35). In preparing this manuscript we adhered to the CORE-Q checklist for qualitative research reporting (36).

### Sampling and Recruitment

Persons were eligible to participate if they were aged 18 years or older; reported having a TBI diagnosed by a HCP (including concussion); were at least 6 months post-TBI; and who self-identified as having low mood at any point post-TBI, having understood that this study was examining healthcare experiences of adults with TBI and depression. A depression diagnosis was not required because depression may have been overlooked in this population by HCPs. Recruitment was by convenience sampling followed by snowball sampling. Individuals saw a physical or online flier (posted around the University of Toronto campus and on websites/mailing lists of organizations serving persons with TBI and/or depression, e.g., Ontario Brain Injury Association). Recruitment continued until we were confident we had answered the research question (37) and achieved rich understanding of patient perspectives on the phenomenon (38).

### Data Collection

After obtaining informed, verbal, and written consent from participants, data were collected by the first author through a semi-structured interview lasting ~1 h. The first author is a HCP experienced in qualitative research and in working with individuals with TBI; she conducted this study as part of her graduate degree requirements. The interviewer and participants had no prior relationship. Each interview was conducted one-on-one by phone, with the interviewer and participant at private locations due to COVID-19 restrictions prohibiting in-person meetings (March-May 2020). Interviews were audio-recorded and based on an interview guide with six questions and probes regarding participants' post-TBI healthcare experiences, impact of low mood, what was considered important in TBI recovery. These questions were reviewed with the research team and piloted with the initial three participants to enhance its appropriateness, clarity, and rigor of data collection (39); no changes were deemed necessary. The interviewer made field notes to capture contextual information such as participant behavior and notable responses. Audio recordings were transcribed verbatim using an independent transcription service and anonymized prior to analysis. Data analysis coincided with data collection to enhance its depth and quality (40).

### Data Analysis

We followed Braun and Clark's (41) six-staged thematic analytic approach to identify patterns across participant responses: familiarizing with data, generating initial codes, searching for themes, reviewing themes, defining and naming themes, and producing the report. We derived coded categories by adhering to the “surface of the data and events” (42) without need for in-depth interpretation. The initial three transcriptions were coded independently by two coders (AC with KND who is a qualitative expert) using NVivo software. They consensually arrived at a coding scheme which included definitions and application rules. The first author then applied the coding scheme to the remaining interview transcripts, creating new codes and disambiguating as needed before reviewing with the other coder. Themes were data-driven without use of preconceived categories. Initial themes and a preliminary thematic map were presented to the research team for discussion. The same two coders (AC, KND) then reviewed the feedback and independently refined the thematic map to reach coherence before a final discussion with the research team confirmed the final thematic map.

## Results

**Figure 1:**
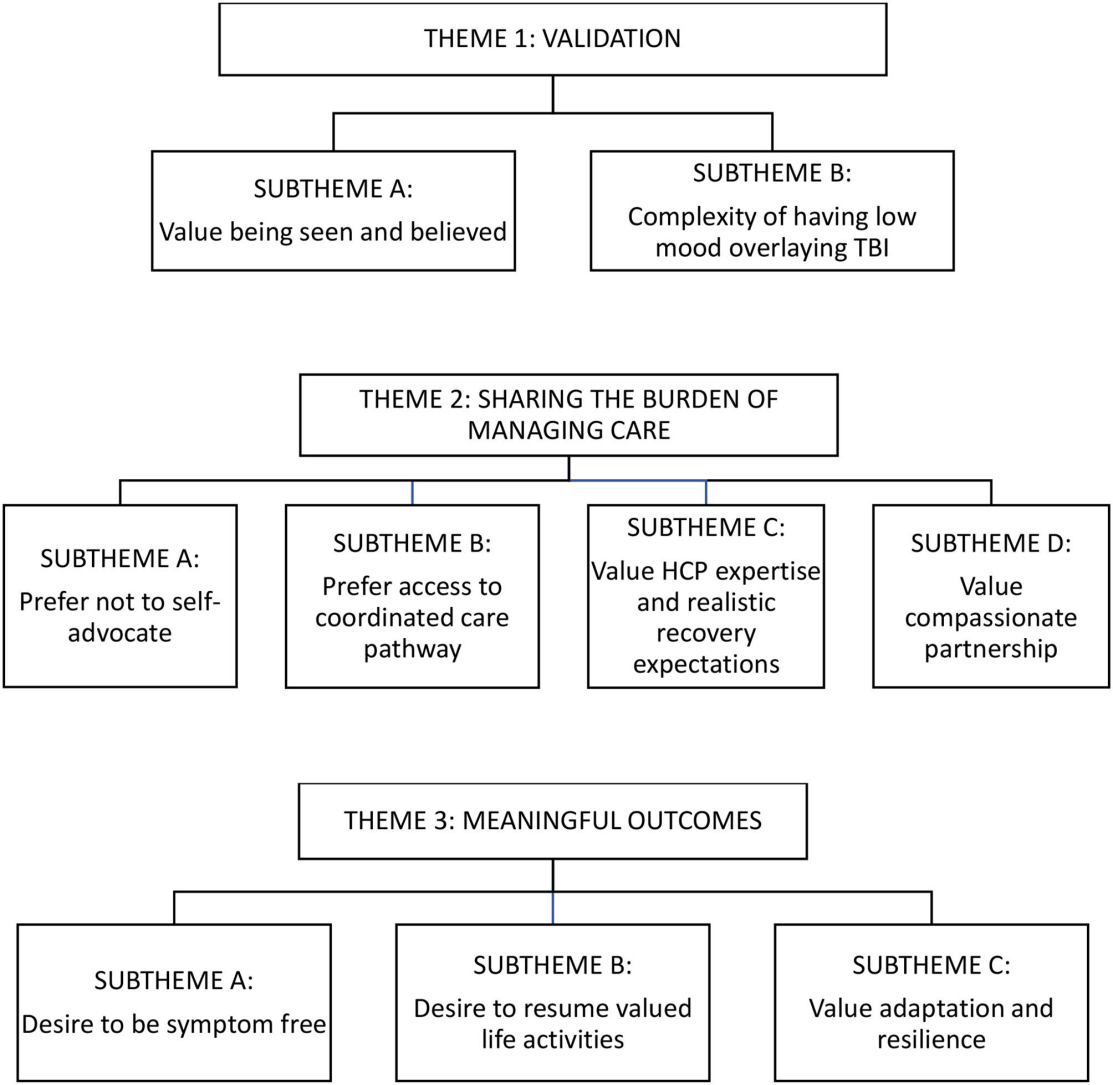
Thematic map.

Thirteen participants shared their experiences of healthcare post-TBI and their perspectives on values, preferences, and desired outcomes. All individuals screened were eligible and none refused to participate. Participants ranged in age from 30 to 58. Most were female, had sustained a concussion or mild TBI (self-reported term of diagnosis), and all but one were from the province of Ontario, Canada (Table 1). Three themes were identified: “validation,” “sharing the burden of managing care,” and “meaningful outcomes.” Subthemes are elaborated below with key illustrating quotes. Figure 1 shows a thematic map of the themes and subthemes.

**Table 1:** Participant demographics.

**Participant**	**Age at screening (years)**	**Sex**	**TBI severity (most recent TBI)**	**Lifetime number of TBIs**	**Time since TBI (months)**
H	32	Female	Mild TBI	2	30.8
U	44	Female	Concussion	1	7.6
F	38	Female	Moderate TBI	3	41.6
T	27	Female	Concussion	1	9.2
N	37	Female	Mild TBI	2	52.4
D	41	Female	Mild TBI	1	44.3
B	41	Female	Mild TBI	1	25.1
A	58	Female	Mild TBI	3	14.9
S	31	Female	Concussion	1	30.2
M	30	Female	Concussion	1	18.2
Y	48	Male	Concussion	1	44.6
R	42	Female	Concussion	1	14.4
C	58	Female	Concussion	8	41.8
	Mean (SD): 40.5 (9.8) Range: 30-58			Median: 1 IQR: 1	Mean (SD): 28.9 (15.3) Range: 7.6-52.4

*IQR, interquartile range; SD, standard deviation; TBI, traumatic brain injury*.

### Validation

Participants expressed that their experience of TBI recovery was very much based upon receiving validation for their health conditions from HCPs. They described their conditions of concussion/mild TBI and depression as invisible disabilities and emphasized that low mood following TBI is complex and that both conditions should be understood together.

#### Value Being Seen and Believed

Participants experienced challenges when they felt their concussion/mild TBI symptoms and mood struggles were not acknowledged or taken seriously; their experiences were often doubted and resulted in their conditions being un- or under-treated as many struggled to access care. Participants expected HCPs to provide proper care without second guessing the truth of their experiences:

*I think first and foremost is being validated… to just immediately be believed is worth its weight in gold. (Participant D)*

Validation by HCPs was critical because some participants had to deal with insurance companies that questioned the veracity of their concussion symptoms, which was exacerbated by the fact that concussions are invisible brain injuries:

*There was nothing showing me what this brain concussion is. Like they did an MRI to make sure there's no structural damage but like there's nothing that shows. (Participant S)*

Participants perceived that insurance companies wanted to deny their legitimate care claims by insurers' constant suspicion that they were “*working the system”* (Participant T), which further deteriorated their mental health:

*I didn't feel at all like I had anxiety or depression at the beginning, and then as I like no one helped me and the pain got worse, the pain got terrible, then I started getting like suicidal and not wanting to be here because it felt like well then you see all the car insurance doctors and they're just like no there's nothing wrong with you. You're fine. You're crazy, and then you start thinking okay am I crazy? Like am I making up this pain in my head?... And then it's just a whole rabbit hole you go down where you just doubt yourself. (Participant B)*

Participants indicated that resistance from insurance companies was to be expected, but they expected HCPs to validate that the symptoms they disclosed were real:

*At the end of the day doctors have a responsibility to be reflective practitioners and if they're not going to do that, I can't make them. It really annoys me when I'm not believed or when it's like, oh you're exaggerating because you want more money, you know. Like all the money in the world isn't gonna give me what I lost from this. Like it's just don't add insult to injury you know. (Participant D)*

In addition, some participants encountered HCPs who did not understand that concussion symptoms can persist. Instead, HCPs attributed their ongoing physical and cognitive symptoms as manifestations of mood or anxiety complaints:

*Like I had to give proof to my healthcare providers that I still have concussion symptoms even when my mood is good. Like it's they're searching out ways to find a way to say that it's just anxiety despite having physical symptoms and despite being able to not read… it felt a lot of the times like a fight against people who from the outset kinda didn't wanna believe me. (Participant M)*

When HCPs validated their concussion/mild TBI symptoms, even in the presence of mental health issues, participants felt understood and hopeful:

*For people who got me, I guess they more made me feel validated and made me feel like I wasn't crazy, even if I'm having mental health issues... it's validating finding someone [a doctor] who understands and so they know what you're going through and these are the steps. They give you hope. (Participant B)*

#### Complexity of Having Low Mood Overlaying TBI

The low mood described by participants seemed to result from a culmination of factors, such as wondering whether HCPs were truly on their side and whether they would ever recover from their concussion/mild TBI.

*I feel like I'm in limbo. I feel like I don't know what's gonna happen… you got people who have been dealing with the same thing for ten years. That's pretty depressing. (Participant R)*

The lack of certainty surrounding their TBI experiences was exacerbated by participants' feeling that their grief and intangible losses often went unacknowledged:

*Because no one, no one in life likes to talk about grief or loss… people assume grief and loss is supposed to be when somebody dies. They forget intangible loss like when you know you had a potential to get promoted within your career and it just goes away… over time I think it gets compounded. And when we think major events like a brain injury or some sort of health concern happens, it just all kinda weighs down on you. (Participant F)*

Participants further lamented the uncertainty stemming from barriers to healthcare access including long wait times to see specialists:

*[The doctor] waits till you're past six months before you're referred and then you have to wait four months. I don't know. I just wonder if things could have been done earlier and differently. You know so I would not be where I am now. (Participant A)*

*That concussion care is insufficient really amplified the low mood whereas… if [resources] were in place from the beginning, like you don't get as much of that hopeless kind of nothing's gonna be resolved thing. (Participant S)*

Others described the compounding effect from dealing with multiple factors and the need for HCPs to understand, validate, and normalize the complexity of having low mood post-TBI:

*I know concussion can trigger anxiety and depression but then how much of it is situational and if you don't have someone who understands different aspects of that situation, how can that really help you? (Participant R)*

*Before asking about somebody's mood, to say it's really normal for people with concussion or TBI to be experiencing low mood because of the TBI, because of all the circumstantial things that are happening. So it's, it's not that there's something wrong with you or that you're doing things wrong or that we're gonna use it against you, if you do have a low mood. (Participant M)*

### Sharing the Burden of Managing Care

Participants expressed feeling the brunt of responsibility for recovery but preferred having a standardized and coordinated care pathway for concussion rather than constantly self-advocating for care. They identified how managing care would be eased if HCPs practiced two specific values: setting realistic recovery expectations and offering compassionate partnership.

#### Prefer Not to Self-Advocate

Out of necessity, participants self-advocated for resources and healthcare access since their invisible conditions were not always believed; they sought referrals to care in the absence of HCP-initiated referrals. Many participants expressed feelings of abandonment by HCPs and the healthcare system and that their growing desperation affected their mood:

*You're starting in a situation where okay you've got low mood. Well then try and get help when people don't believe you… I was not my usual self, right. Like I wasn't able to advocate properly. I just, I was so vulnerable… It was like as snowball situation when you're rolling a snowball down the hill and you start with one problem but then because it goes untreated or it gets treated in the wrong manner, it's now this big honking snowball. (Participant D)*

While experiencing debilitating symptoms, self-advocacy seemed challenging and demoralizing to participants:

*You're just floundering around… Like it's just a terrible, it's an awful thing to put the person with the brain injury in charge of figuring out how to make themselves better. (Participant A)*

#### Prefer Access to a Coordinated Care Pathway

Some participants became wary of the healthcare system due to their “*experience with healthcare providers and with trying to seek out care [being] traumatic in a way“ (Participant M)*. Others relied on external sources such as the local brain injury association to link them with service resources but would have preferred having a “*disease pathway” (Participant U)* so that access to concussion care would not be haphazard, delayed, or outright denied:

*It would just be really helpful if there was a more integrated system and treatment and more resources… and like I guess case management… there are a lot of resources out there but they're hard to find. (Participant N)*

A coordinated care pathway could incorporate what participants termed a “*project manager because… I didn't have the cognitive ability to like figure it all this stuff” (Participant F)*. Another participant believed having care coordination would have lessened the negative effects on mental health:

*Getting just all the paperwork and responding to the same type of questions over and over again, reliving it just caused much more anxiousness and anxiety and led to depression. And if there was just a more common thread of a person or organization that could just take care of that, I guess could have helped. (Participant Y)*

#### Value Healthcare Provider Expertise and Realistic Recovery Expectations

Given their perplexing experiences, participants expect HCPs to be “*up to date on concussion recommendations” (Participant M)*, preparing them with realistic recovery expectations including the possibility of postconcussion syndrome.

*I would have loved for my nurse practitioner not to have said you'll be better in seven to ten days. Even just to say the majority of people get better, eighty percent of people get better in seven to ten days, we can hope for that but here's what we're gonna do if you happen to fall into that last twenty percent. (Participant M)*.

Without realistic recovery expectations and awareness of postconcussive syndrome, some participants described blaming themselves for their ongoing symptoms, which affected their mental health:

*I wish somebody had told me that it'd be worse than anything you've ever experienced and other people have had this experience and it's normal… because treating somebody like they're the first person that's ever said I don't feel like the same person anymore doesn't help. Right like it would have gone a long way to normalizing my experience in making me not feel like a headcase… ‘Cause I really beat myself up for not getting better. (Participant D)*

#### Value Compassionate Partnership With Healthcare Providers

Participants appreciated when HCPs supported their recovery journey with “*soft skills… Are they listening to the person or are they just looking the person as off, off their diagnoses?” (Participant T)*—skills beyond technical competencies. These soft skills pertained to the quality of HCP-patient interactions supporting person-centered care.

*So the first OT (occupational therapist) that I was given was sent by [the insurance company]. This woman doesn't know her ass from elbow quite frankly and was not looking out for my best interest at all right. [But] the OT from the neuroplasticity clinic is working for me, right. So I think that makes a real difference… you've got somebody who at the core of her whole values system is the patient... not the bottom line, not money, not whatever. (Participant D)*

Participants valued when HCPs gave hope for their recovery and were open to trying their suggestions. They also valued when HCPs elicited details of their mood in a caring way:

*For the low mood, like if they were dealing, there's probably a lot of people that are dealing with stuff that they just hide and suppress and push down. It's important to gently, really in a caring way figure out how you can pull out that information from them so you can actually help them move past that old stuff and help them process this new stuff. (Participant F)*

### Meaningful Outcomes

Participants revealed three categories of desired outcomes. They wanted to be symptom free and to resume valued life activities. They had also come to value their resilience and ongoing adaptation as an unexpected outcome to the challenging experiences of recovery:

*I sort of get the idea that you don't expect to go back to the way it was. You go back to a new normal. (Participant A)*

#### Desire to Be Symptom Free

Participants want their persisting cognitive and mental health challenges to be resolved since it affects their ability to live satisfying post-TBI lives.

*It's really critical to treat mood issues obviously early, and I think aggressively would be nice too. I think it's important for outcomes. I think it's important for quality of life while we're going through some of the darkest times. (Participant H)*

*The cognitive stuff has been worse than the physical stuff, even though the physical stuff has been terrible cause I feel like you know you can do things to help some of that go away, but the cognitive stuff I just, I can't seem to move forward with it very well. (Participant R)*

Participants described the importance of addressing both cognitive and mental health symptoms so that the journeys toward being symptom free were in tandem.

*Your brain cannot heal until you deal with some of the mental health side of things… your brain needs that in order to heal from the concussion. (Participant A)*

*Just having some sort of mental support that knows about concussions and stuff would have made a big difference I think. (Participant B)*

When participants sought help for mental health, they preferred treatment choices like psychological care in addition to pharmacological treatment, especially if they perceived that their low mood is a function of grief:

*Oh well just go on this anti-depressant this medication and that will help. And that's not necessarily the best approach to take it. Such a complex issue that you're working with and really, everyone who has postconcussive syndrome really needs a psychologist who knows about concussion because it's very complex in nature. (Participant T)*

*I am not depressed. I think I'm appropriately sad given the circumstances and the amount of things that I've lost, and my grieving process. I do not want any sort of antidepressant… [it] highlights the gap in the system… nobody is helping us grieve. (Participant F)*

####  Desire to Resume Valued Life Activities

Participants gave the impression that they experienced TBI and depression as interruptions to life activities including relationships; they attributed their withdrawal from social interactions to their debilitating symptoms and to how people do not understand invisible disabilities:

*That's been the biggest challenge. I haven't been able to like the social piece, whether it's being at home with the kids or with my family or being in group settings, that part is very very challenging. (Participant S)*

*I wanna be active and healthy and see people again. It was really lonely. I don't know. I just, I don't even care. That's what I feel like. I don't even care if all the cognitive comes back. (Participant R)*

Resuming valued life activities included supporting others out of their experiences, being able to “*add value to society” (Participant F)* by “*[doing] meaningful work again” (Participant M)* and pursuing their life goals.

*I still want a meaningful career. I'm hoping to start a family actually from coming off of a lot of my, all of my migraine drugs. In anticipation of that going to try and start a family and yeah live as best I can with the ongoing symptoms. Yeah, I really look forward what's ahead, acknowledging that it will look very different. (Participant H)*

#### Value Their Adaptation and Resilience

However, many participants seemed to recognize over time that they might not return to their previous level of functioning but that they must find a way to continue.

*I've accepted a lot of things and I am moving forward as opposed to just being stuck which is what happened very quickly and where the depression and anxiety really kicked in. (Participant Y)*

Describing this process, other participants preferred the term “adaptation” over “acceptance” because it better reflected the need for continual adjustment:

*I don't know that there is an end to recovery first of all. I imagine that there's continued adaptation. Yeah I don't think there's an end. But I hope, what I hope for is continued, like I hope for continued vitality. (Participant N)*

For some participants, ongoing adaptation bred resilience from dealing with debilitating symptoms and struggles to access care. Others attributed innate qualities such as self-motivation which supported their resilience:

*You get knocked down every second step you take and you have to just keep going. And yeah I've got quite a bit of resilience and perseverance especially because I continued to have mental challenges unfolding, right. So being able to just keep at it was something. (Participant H)*

Resilience seemed to contribute to their personal growth via having a different perspective on life post-TBI.

*I want to enjoy my life now… there's a recognition of the finite element of life and wanting to recognize the priorities have shifted, my perspectives have shifted. Which is helpful with the mood, recognition of that and what do I wanna do, what do I wanna spend or waste my time on. (Participant Y)*

## Discussion

To our knowledge, this is the first qualitative study to recruit a TBI sample with self-identified low mood. The results of this study describe their values, preferences, and desired outcomes for person-centered rehabilitation, broadening our understanding of how better person-centered care from HCPs and the healthcare system can support participation.

Most participants reported having concussion/mild TBI so there were few physical markers of their injuries and their mood symptoms were self-reported. When HCPs did not acknowledge their conditions, symptoms, and functional impact, they felt invalidated and struggled to access care. This sense of invalidation when disbelieved by HCPs has been reported in the literature [e.g., (43)] particularly among those with postconcussive syndrome where symptoms last beyond 6 months post-injury (44). This study extends our understanding of the impacts of invalidation: patients must consequently advocate for themselves despite preferring HCPs to share the burden of managing care. Further research is needed to understand the role that validation plays in the HCP-patient relationship within TBI rehabilitation, including the extent to which HCPs inadvertently or purposefully invalidate and how. The latter requires knowing the perspectives of HCPs which was outside the scope of this paper. One possible explanation, though, is that the degree to which conditions are invisible can negatively influence how others behave toward the person with the disability (45, 46).

Support for this concept can be inferred by comparing our study with the findings of a qualitative metasynthesis examining what influences patient-HCP interactions in physical therapy (47). The values expressed by participants closely match three of four metasynthesis findings: therapists' expertise, communication skills, and person-centered care. The codes belonging to the fourth finding of “organizational and environmental factors” (47), “time with HCPs” (enough, unrushed) and “flexibility with appointments and care” (HCPs being accommodating), were not reflected by most of our participant perspectives. However, many did express similar frustration with *accessing* care appointments—contingent upon validation, which may precede *flexibility* with appointments. Ergo, *access to care* could be considered a superordinate organizational factor at the healthcare system level: promoting downstream participation by having a coordinated care pathway for concussion/mild TBI could mitigate the access challenge and is discussed below. Interestingly, validation was not identified in the metasynthesis perhaps because included studies focused on musculoskeletal physical therapy which may tend to be more visible.

Our participants emphasized the nature of interactions with HCPs and expectations for them to validate issues and concerns. Other studies support that individuals with an invisible disability assess the quality of HCP interactions by how well they are “seen, heard, and believed” (48). In our sample, feeling disbelieved seemed to pose the risk of compounding emotional distress—especially when they felt HCPs dismissed their cognitive symptoms as complaints because of mood or anxiety. This disbelief appeared to increase feelings of uncertainty regarding recovery, as is reported elsewhere (49). In further support, some participants described worsened mood when led by HCPs to believe that they should have recovered yet had not; many blamed themselves for the lack of progress and feared they would never recover.

Conversely, other participants felt that HCPs gave them hope because their interactions were person-centered and that HCPs cared about their mental health. From these accounts, HCP-patient interactions seem to have a negative or positive effect on recovery expectations. Therefore, intervening to change HCP behaviors may improve patient experiences. For example, to counter the stigmatization of invisible disabilities, strategies have been proposed to reduce the negative attitudes of HCPs toward patients with mental illness (50). Other literature demonstrates that HCPs and patients often differ on beliefs about illness and its meaning (51) and on indicators of “successful” recovery (52). Setting realistic recovery expectations influences health outcomes including depression (53–55). Therefore, HCPs should provide credible information to cultivate hope for recovery (43). During rehabilitation, the onus is on HCPs to seek alignment of recovery expectations with outcomes or at least address gaps between these (49) and, coherent with the results of this study, to address any “recovery disappointment” (54) that may arise.

Refined person-centered care approaches could be applied for individuals with TBI and low mood. Firstly, while clinical guidelines recommend reassuring individuals of expected recovery within 2 weeks of their concussion/mild TBI (56), HCPs must recognize that they are shaping patients' expectations from the earliest interaction. It would be advantageous for HCPs to prioritize assessing and addressing what patients believe are important (47, 53) such as the desired outcomes for participation identified in this study. By focusing on patient priorities, HCPs are supporting person-centered care which has been shown to improve treatment outcomes, satisfaction with care, and quality of life (14, 15).

Secondly, providing person-centered care can include enabling clients to interpret their symptoms, their attached meaning (57), and any (lack of) progress in recovery. Recovery expectations can be shaped through use of motivational interviewing (58) which is person-centered and validating (49). Thirdly, a goal-setting approach focused on patient-important outcomes can decrease emotional distress and improve mood (59). With person-centered goal setting, progress can be monitored toward meaningful goals and recovery expectations be discussed in that context. For example, if the rehabilitation goal has not been amenable to change, then this feedback can be used to modify the HCP's approach or to adjust the patient's recovery expectations. Having agreed-upon, realistic expectations about the rate of goal progression may buffer against low mood, which has been interpreted as a response to goal failure (60).

Therefore, HCPs can foster patients' “*new normal” (Participant A)* and self-identity to be one that comprises both loss and continuity/growth (61); doing so will enable appropriate recovery expectations and hope, factors identified by participants in this study and in others as critical elements in rehabilitation (29, 52). So while individuals with TBI may feel loss and grief that may not have an endpoint (62), HCPs can support their psychosocial adaptation by acknowledging their “*hope for continued vitality” (Participant N)*.

For our participants, preferences were driven by perceptions that the healthcare system overlooks concussion/mild TBI, such as feeling that their cognitive and mental health needs were inadequately addressed. Ongoing symptoms affected their life participation, notably their ability to re-engage socially and to return to work. Participants described having to self-advocate, lamenting how resources and services were not organized for their optimal recovery. Significant system-level changes to reduce barriers to participation may be needed to support rehabilitation. To reduce the burden of managing care, participants preferred having a coordinated care pathway, a standardized, fulsome series of services arranged for everyone with a concussion/mild TBI.

An extended recovery pathway could reassure individuals with postconcussive syndrome that they would have ongoing access to rehabilitation. Further, a TBI pathway can include recovery expectations and timeline (43). Care pathways that ensure rehabilitation services during key transitional periods, such as that leading to resumption of life activities, could counter the typical experiences of waiting lists and lack of psychological support (63). Establishing a pathway with these described features would be a substantial step toward promoting participation by providing better person-centered care for adults with concussion/mild TBI and low mood.

### Limitations

We recruited by self-reported low mood instead of diagnosed depression, opting to include individuals whose mental health challenges might have been missed by HCPs. Subsequently, this sample may have a significant range in depressive symptomology affecting their expectations of HCP interactions. Due to our sampling strategy, our sample may not be fully generalizable: most participants were female with concussion/mild TBI. This “mild” severity of TBI and prolonged symptomology heightens the likelihood of their conditions being experienced as invisible and invalidating. The presented data is, however, consistent across participants and with data from other studies examining invisible conditions. Women are also more likely than men to participate in research studies and to be depressed (64). Women tend to seek healthcare services more than do men, but there is not a significant gender difference in consultation rate for depression (65). The male participant's responses were consistent thematically with those of female participants, except that difficulty accessing post-TBI care may be partially attributable to his initial reluctance in recognizing available supports. Since interviews were conducted by phone, subtle shifts in mood and behaviors might have been missed which could have yielded different answers. However, participants seemed to require little prompting to provide rich descriptions of their experiences as insiders (66). Participant bias is likely as they were motivated to share their perspectives, but this is the aim of qualitative research. Without member checking, we do not know whether the thematic map resonates with all participants, but multiple participant quotes were readily obtained for each subtheme.

## Data Availability Statement

The original contributions presented in the study are included in the article, further inquiries can be directed to the corresponding author.

## Ethics Statement

The studies involving human participants were reviewed and approved by Office of Research Ethics at the University of Toronto, Canada. The participants provided their written informed consent to participate in this study.

## Author Contributions

AC, HC, and KND contributed to the conception, design, and methodology of this study. AC and KND performed the formal analysis. HC and DRD supervised this study. AC wrote the first draft of the manuscript. AC, KND, BK, DRD, and HC reviewed and edited the manuscript. All authors contributed to manuscript revision, read, and approved the submission.

## Funding

AC received support from the Peterborough K.M. HUNTER Charitable Foundation Graduate Award and the Queen Elizabeth II/Patty Rigby and John Wedge Graduate Scholarship in Science and Technology during her doctoral studies.

## Conflict of Interest

The authors declare that the research was conducted in the absence of any commercial or financial relationships that could be construed as a potential conflict of interest.

## Publisher's Note

All claims expressed in this article are solely those of the authors and do not necessarily represent those of their affiliated organizations, or those of the publisher, the editors and the reviewers. Any product that may be evaluated in this article, or claim that may be made by its manufacturer, is not guaranteed or endorsed by the publisher.
